# Mortality Audit of Cancer Patients in the Department of Medical Oncology at a Tertiary Cancer Care Centre in South India

**DOI:** 10.7759/cureus.56296

**Published:** 2024-03-16

**Authors:** Akansha Choudhary, Linu A Jacob, Suresh Babu, Lokesh K.N, Rudresha A.H., Rajeev L.K., Smitha Saldanha, Tarjina Begum

**Affiliations:** 1 Medical Oncology, Kidwai Memorial Institute of Oncology, Bangalore, IND

**Keywords:** haematological cancer mortality, cancer deaths, solid cancer mortality, south india, mortality audit

## Abstract

Considerable advances in the diagnosis and treatment of cancer have made a huge impact on morbidity and mortality from neoplastic diseases. However, cancer remains the leading cause of death across the world. This is a retrospective study carried out at a tertiary cancer care centre (Kidwai Memorial Institute of Oncology, Bangalore) in South India. Case records of all cancer patients who died while receiving inpatient treatment between January 2022 and December 2022 under the Department of Medical Oncology were reviewed and studied. There was a total of 240 deaths. Out of these, the majority of deaths 147 (61.25%) were patients with haematological malignancies while the remaining 93 (38.75%) were patients with solid tumours. In patients with solid tumours, the majority 49 (52.7%) were in the age group of 40 to 60 years while only 18 (19.35%) patients were less than 40 years. The majority of patients were male sex i.e. 55(59.1%) and undergoing treatment with palliative intent 81 (87%). The most common organ was the lung in 21 patients (22.6%) followed by the breast while the most common system involved was the gastrointestinal tract in 28 (30.1%) patients. The most frequent cause of death was progressive disease in 72 (77.4%) while sepsis (11 patients; 11.8%) was the second most frequent cause of death in solid tumours. In haematological malignancies, also a significant number of 57 (38.8%) patients were in the age group of 40 to 60 years. Fifty-two (35.3%) patients were in the age group of 22 to 40 years. The majority were male sex (79 patients; 53.7%). About the phase of treatment, the majority of deaths 45 (30.6%) were during induction and under evaluation. Those with relapse/refractory disease were 38 (25.9%). A substantial number of patients had acute myeloid leukaemia 47 (32%) and five (3.4%) deaths were acute promyelocytic leukaemia patients. Twenty-three patients (15.6%) had acute lymphoblastic leukaemia. The most common cause of death was sepsis in 76 patients (51.7%) while intracranial bleeding was in 34 patients (23.1%). In some patients, there were multiple causes leading to death. Mortality audits are important to evaluate the services being provided at any centre. One can appreciate the lacunae in handling a particular disease or flaws in a treatment protocol or the staff delivering the treatment. Sepsis is the leading cause of death in patients with haematological malignancy; even in solid malignancy sepsis accounts for a substantial proportion of deaths and should be handled aggressively to save lives.

## Introduction

Considerable advances in the management of cancer have made a huge impact on morbidity and mortality from neoplastic diseases. Malignant neoplasms are, still, the leading cause of death all over the world, responsible for nearly 10 million deaths according to the Globocon 2020 data [1]. A significant number of patients with cancer die, while on active anti-cancer therapy. A mortality audit gives important information into the trends of death and cause of death and may identify the remedial steps and suggest measures to reduce mortality. In the early 1980s, a confidential review of mortality with anaesthesia was set up in the United Kingdom, mentioned by Lun and Mushin in 1982 [2] which later in 1988, evolved into National Confidential Enquiry into Perioperative Deaths (NCEPOD) and includes Medical and surgical deaths [3]. A large majority of mortality data in oncology is in the form of published clinical trial results which pertain to specific cancers. We analysed the mortality data of inpatient deaths at our centre with an aim to identify the number of deaths occurring in patients undergoing treatment with palliative and curative intent and if deaths could have been prevented by commission or omission of some action or intervention.

## Materials and methods

This is a retrospective study carried out at a tertiary cancer care centre (Kidwai Memorial Institute of Oncology, Bangalore) in South India. Case records of all cancer patients who died while receiving inpatient treatment between January 2022 and December 2022, under the Department of Medical Oncology were reviewed and studied. Our tertiary cancer care centre in South India has various departments medical oncology, radiation oncology, surgical oncology, head and neck oncology, gynaecology, oral oncology, pediatric oncology, and pain and palliative care providing outpatient and inpatient services. All inpatient deaths in the institute are recorded in the mortality register and amongst these, all the deaths of patients from the Medical Oncology Department are discussed in a monthly mortality meeting. The study was designed with the aim of identifying the number of deaths occurring in patients undergoing treatment with palliative and curative intent and if deaths could have been prevented by the commission or omission of some action or intervention. The primary objective of this study was to identify the cause of death among patients admitted under medical oncology in the medical ICU, medical wards and emergency room. The secondary objective was to study the phase of treatment intent and the presence of comorbidities. The study included all those patients who died while receiving inpatient treatment between January 2022 and December 2022, under the Department of Medical Oncology, while excluding deaths declared by other departments and deaths at home or deaths of patients who had taken Discharge Against Medical Advice and expired outside the hospital. Descriptive analysis was done for the patient, disease and treatment characteristics. Categorical variables were summarised by frequency. Informed consent was taken from patient relatives. Since none of the patient identities were disclosed in the data, confidentiality was preserved. The protocols adhered to the 2013 revision of the Helsinki Declaration of 1964 and the ethical guidelines set forth by the competent committee on human testing.

## Results

In a year from January 2022 to December 2022, there were a total of 1986 new registrations in the department. Out of these, 1168 were males and 818 were females. The total number of patients attended to was 72134, including new registration, follow-up visits, and referrals to the outpatient department. The number of in-patient admissions was 3050 males and 2272 females, making a total of 5322 admissions. There were 240 deaths in total with 134 and 106 among males and females respectively. The ratio of death to hospital admissions was 4.5%. Among males, the same ratio was 4.39% while it was marginally higher among females at 4.66%. Of the total deaths, patients with haematological cancers accounted for 147 (61.25%) of the deaths, while patients with solid neoplasms accounted for the remaining 93 (38.75%) deaths reflecting an increased admission of haematological malignancies at our hospital. Only one patient was on best supportive care as the patients undergoing best supportive care are usually transferred to the pain and palliative care department at our hospital. Ninety-one patients undergoing palliative intent treatment were coded for the Do Not Resuscitate (DNR) protocol. Separate analyses of the data were carried out simultaneously for solid (Table 1) as well as haematological cancers (Table 2).

**Table 1:** Characteristics of 93 Patients With Death From Solid Malignancies GI, gastrointestinal; GU, genitourinary

Variable	Number (percentage)
Age Group (years)	
<20	1 (1.1%)
20-40	17(18.3%)
40-60	49 (52.7%)
>60	26 (28.0%)
SEX	
Males	55 (59.1%)
Females	38(40.9%)
Intent Of Treatment	
Definite	12 (12.9%)
Palliative	81 (87%)
Diagnosis (Primary Cancer Site)	
Breast	18 (19.4%)
Lung	21 (22.6%)
Head and Neck	8 (8.6%)
GI	28 (30.1%)
GU	5 (5.4%)
Sarcoma	4 (4.3%)
Gynaecological	4 (4.3%)
Others	9 (9.7%)
CAUSE OF DEATH	
Progressive disease	72 (77.4%)
Sepsis	11 (11.8%)
Respiratory failure	7 (7.5%)
Tumor bleed	2 (2.2%)
Others	1 (1.1%)
Comorbidities	
Present	24 (25.8%)
Absent	69 (74.2%)

**Table 2:** Characteristics of 147 Patients With Death From Haematological Malignancies. AML, acute myeloid leukaemia; APML, acute promyelocytic leukaemia; ALL, acute lymphoblastic leukaemia; CML BC, chronic myeloid leukaemia in blast crises; CML CP, chronic myeloid leukaemia in chronic phase

Variable	Number (percentage)
Age Group (years)	
<20	14 (9.5%)
20-40	52 (35.3%)
40-60	57 (38.8%)
>60	24 (16.3%)
SEX	
Males	79 (53.7%)
Females	68 (46.3%)
Phase Of Treatment	
Induction	45 (30.6%)
Consolidation	1 (0.7%)
Under Evaluation	45 (30.6%)
Palliative	10 (6.8%)
Reinduction	2 (1.4%)
Relapse/ Refractory	38 (25.9%)
Others	6 (4%)
Diagnosis	
AML, APML	47 (32%), 5 (3.4%)
ALL	23 (15.6%)
CML BC, CP, under evaluation	4 (2.7%), 2(1.4%),1(0.7%)
Multiple myeloma	3 (2%)
High grade B cell lymphoma	8 (5.4%)
T cell lymphoma	3 (2%)
acute leukaemia under evaluation	23 (15.6%)
Lymphoma under evaluation	22 (15%)
Others	8 (5.4%)
Cause Of Death	
Intracranial bleed	34 (23.1%)
Sepsis	76 (51.7%)
Tumor Lysis Syndrome	22 (15%)
Progressive disease	9 (6.1%)
Respiratory failure	8 (5.4%)
Renal failure	5 (3.4%)
Cardiac failure	6 (4.1%)
Comorbidities	
Present	25 (17%)
Absent	122 (83%)

Solid tumours

A greater number of deaths with solid neoplasms occurred in male patients i.e. 55 deaths (59.1%). Amongst solid neoplasms, there was no death in early stage, and 12 (12.9%) deaths occurred in patients with locally advanced disease while the remaining majority were metastatic disease. An overwhelming majority of 81 patients (87%) were receiving palliative treatment. Hardly, 18 (19.35%) individuals were younger than 40 years old. 49 cases (52.7%) were in the 40-60 age range. A large proportion of deaths in young patients probably is an indirect reflection of the lesser number of elderly patients undergoing active treatment at our hospital. There may be multiple reasons for this like elderly are often neglected in the family and are not given adequate medical care, treated in a nearby hospital rather than being taken to a far-off tertiary cancer centre, or allocated best supportive care due to poor tolerance or poor support from family. In 21 (22.6%) patients, the most frequent primary organ affected was the lung, followed by the breast. In 28 (30.1%) patients, the most common system affected was the gastrointestinal tract. The most frequent cause of death was progressive disease in 72 (77.4%) while sepsis in 11 (11.8%) was second most common cause of death in solid tumors. Sepsis included patient admitted with sepsis there was hardly any case of hospital acquired infection. A substantial number of patients 69 (74.2%) did not have any comorbid illness. The comorbidities listed in the study were diabetes, hypertension, bronchial asthma/chronic obstructive pulmonary disease, thyroid disorders, chronic renal disease, coronary artery disease, congenital heart disease, tuberculosis, hepatitis B, and retroviral disease.

Haematological cancers

In hematological cancers also, the majority 57 (38.8%) cases were in 4th to 6th decade of life. Another 52 (35.3%) patients were in the age group of 20 to 40 years. The greater proportion were from male sex 79 (53.7%). About the phase of treatment, a larger number of deaths 45 (30.6%) were during induction and under evaluation each. Those with relapse/refractory disease were 38 (25.9%).

A substantial number of patients were with acute myeloid leukaemia (AML) 47 (32%) and five (3.4%) deaths were acute promyelocytic leukaemia (APML) patients. Twenty-three patients (15.6%) had acute lymphoblastic leukaemia (ALL). The most frequent cause of death was sepsis 76 (51.7%) while intracranial bleeding was 34 (23.1%). Sepsis included both patients admitted with sepsis or hospital acquired infection. In some patients, there were multifarious causes contributing to mortality. No comorbid illness was present in 122 (83%) patients.

## Discussion

To our knowledge, this is the first mortality audit of cancer patients from any centre in South India. In a study on death in cancer patients, carried out in North India by Prakash et al. [4], the majority of deaths at 57% (147/249) were from solid tumours, in contrast to our study. Also, the age group with maximum mortality was 35 to 59 years with 49% deaths similar to our study. Death amongst patients undergoing curative intent treatment for solid tumours was 27. Sepsis was the most frequent cause of death at 45% (118/259) and progressive disease was seen in 25% of all solid as well as hematological cancers. We also found that neutropenia and sepsis played a more significant role in death due to hematological cancers than in solid tumors similar to the study by Prakash et al. Sundriyal et al. also reported 45 deaths in the medical and haematoncology department in another centre in north India, with the majority from solid tumours. Amongst solid malignancies, 23 patients (82.14%) had palliative intent. The most frequent cause of death was sepsis in solid tumours as well as haematological malignancy [5]. 

The median age of inpatient deaths at our centre was 51 years (range 21 to 78 years) similar to the study by Prakash et al. who reported 46 years (range 2 to 83 years) from North India [4], while O'brien et al. reported a median age of 61 years (range 6 to 86 years) [3]. More patients died at a younger age in haematological malignancy than in solid tumours similar to a North Indian study by Sundriyal et al. A majority of patients were males, in the age group of 40-60 years and without comorbidities. This may represent the population that is frequently referred to a tertiary cancer care centre in resource-constrained settings.

O'Brien et al. from Royal Marsden Hospital, UK reported 161 deaths within 30 days of chemotherapy during a six-month period. A vast majority of 77% (124 out of 161) occurred due to progressive disease [3]. In 2000, Bauduer et al. analysed and found that in 70% (56 out of 81 cases) mortality was due to primary progression of the underlying malignancy [6]. Amongst elderly patients, with intermediate or high-grade NHL, receiving CHOP chemotherapy infection was the cause of death in 82% of chemotherapy-related deaths as reported by Gomez et al. in 1998. The chemotherapy-related death rate was 13% [7].

Terminal care practices, mortality reporting practices, hospital admission policies, and patient distribution vary at different centres; hence, there exist differences in reporting the cause of death across the country as well as across the globe. Even at the same centre, the cause of death may be variable with different departments declaring death. Treatment-related neutropenia leading to sepsis, multi-organ dysfunction and death is a leading cause of mortality in oncology patients. However, patients receiving the best supportive care in the palliative care department often have advanced disease as a direct cause of death.

Available literature suggests that chemotherapy was frequently used in the terminal three months. Patients with multiple comorbidities, advanced age, poor performance status (PS), and inadequate response to first-line chemotherapy should be considered for Best Supportive care [8,9]. In our study, only one patient was on best supportive care as the patients undergoing best supportive care are usually transferred to the Pain and palliative care department at our hospital. Ninety-one patients undergoing palliative intent treatment were coded for the DNR protocol. 

In patients who develop neutropenia and sepsis, a few parameters may be helpful to recognise the patients at increased risk of death and therefore start on aggressive management promptly in the form of sending blood and other cultures as well as initiating effective antimicrobial therapy as per the hospital policy. At our hospital, we start third-generation cephalosporin with aminoglycoside. The parameters that may be useful include the type of malignancy- haematological, hypotension, elderly, presence of comorbidities, and previous history of blood culture being positive [10,11].

A major limitation of the study is that it's a single-centre study from a single department. The duration of hospital stay and number of lines of chemotherapy received could not be recorded. The results are subject to bias due to hospital policies of admission and treatment. A substantial number of patients had taken Discharge Against Medical Advice, probably in the last few hours of life and were not included in the study.

## Conclusions

Mortality audits are important to evaluate the services being provided at any centre. One can appreciate the lacunae in handling a particular disease or flaws in a treatment protocol or the staff delivering the treatment. Neutropenia and sepsis play a more significant role in death due to haematological cancers than in solid tumours. More patients died at a younger age in haematological malignancy than in solid tumours. Patients with multiple comorbidities, advanced age, poor PS, and inadequate response to first-line chemotherapy should be considered for Best Supportive care. In patients with sepsis, some factors like haematological cancer, hypotension, elderly, and presence of comorbidities should alert the clinician to start aggressive management promptly in the form of sending blood and other cultures as well as initiating effective antimicrobial therapy as per the hospital policy. This is practised at our centre as well. Whenever any patient with curative intent treatment expires a detailed explanation of events should be prepared and checked for any flaws that can be subsequently improved.
